# Experiences of men who have sex with men when initiating, implementing and persisting with HIV pre‐exposure prophylaxis

**DOI:** 10.1111/hex.13446

**Published:** 2022-04-14

**Authors:** David Gillespie, Fiona Wood, Adam Williams, Richard Ma, Marijn de Bruin, Dyfrig A. Hughes, Adam T. Jones, Zoë Couzens, Kerenza Hood

**Affiliations:** ^1^ School of Medicine, Centre for Trials Research, College of Biomedical & Life Sciences Cardiff University Cardiff Wales UK; ^2^ PRIME Centre Wales and Division of Population Medicine Cardiff University Cardiff Wales UK; ^3^ Department of Primary Care and Public Health Imperial College London London UK; ^4^ Radboud University Medical Center Nijmegen The Netherlands; ^5^ Centre for Health Economics and Medicines Evaluation, Bangor University Bangor Wales UK; ^6^ Policy, Research and International Development, Public Health Wales Cardiff Wales UK; ^7^ Public Health Wales NHS Trust Cardiff Wales UK

**Keywords:** HIV, medication adherence, pre‐exposure prophylaxis, qualitative research, sexual and gender minorities, sexual behaviour

## Abstract

**Introduction:**

HIV pre‐exposure prophylaxis (PrEP) involves the use of antiretroviral medication in HIV‐negative individuals considered to be at risk of acquiring HIV. It has been shown to prevent HIV and has been available in Wales since July 2017. Measuring and understanding adherence to PrEP is complex as it relies on the simultaneous understanding of both PrEP use and sexual activity. We aimed to understand the experiences of men who have sex with men (MSM) living in Wales initiating, implementing and persisting with HIV PrEP.

**Methods:**

We conducted semistructured interviews with MSM PrEP users in Wales who participated in a cohort study of PrEP use and sexual behaviour. Following completion of the cohort study, participants were invited to take part in a semistructured interview about their experiences of taking PrEP. We aimed to include both individuals who had persisted with and discontinued PrEP during the study. The interview topic guide was informed by the ABC taxonomy for medication adherence and the theory of planned behaviour. We analysed our data using reflexive thematic analysis.

**Results:**

Twenty‐one participants were interviewed, five having discontinued PrEP during the cohort study. The developed themes focused on triggers for initiating PrEP, habitual behaviour, drivers for discontinuation and engagement with sexual health services. Stigma surrounding both PrEP and HIV permeated most topics, acting as a driver for initiating PrEP, an opportunity to reduce discrimination against people living with HIV, but also a concern around the perception of PrEP users.

**Conclusion:**

This is the first study to investigate PrEP‐taking experiences incorporating established medication adherence taxonomy. We highlight key experiences regarding the initiation, implementation and persistence with PrEP and describe how taking PrEP may promote positive engagement with sexual health services. These findings may be useful for informing PrEP rollout programmes and need to be explored in other key populations.

**Patient and Public Contribution:**

PrEP users, in addition to PrEP providers and representatives of HIV advocacy and policy, were involved in developing the topic guide for this study.

## 1 INTRODUCTION

By the end of 2020, 37.6 million individuals were living with HIV globally and 690,000 people died from HIV‐related causes.^1^ In Wales, approximately 150 new cases of HIV diagnosed are each year, with 75% of these in men.^2^ While no cure currently exists, advances in treatment, access to testing and treatment services and prevention methods mean that HIV is now a manageable chronic health condition with near‐normal life expectancy.^3^, ^4^ One of the more recent HIV prevention methods is pre‐exposure prophylaxis (PrEP).

PrEP involves the use of antiretroviral (ARV) medication in HIV‐negative individuals considered to be at risk of acquiring HIV (e.g., through high‐risk sexual behaviour or injecting drug use).^5^, ^6^, ^7^ In Wales, tenofovir/emtricitabine (TDF‐FTC) has been licensed as HIV PrEP since July 2017 and can be accessed through National Health Service (NHS) sexual health clinics free of charge by individuals considered to be at risk of acquiring HIV (before this, it was only available through unregulated, online purchase). PrEP is typically prescribed in 90‐day supplies, and both daily (one pill a day around the same time each day) and event‐based (two pills as a single dose 2–24 h before condomless sexual intercourse, followed by one pill a day thereafter until two sex‐free days have passed) regimens are recommended by providers. PrEP users attending clinic to receive their prescription receive an sexually transmitted infection (STI) screen, have their renal function checked and are asked about the sexual history and PrEP‐taking behaviours since their previous visit.^8^, ^9^ The latter aspects of the consultation are pertinent, as ensuring high levels of adherence to PrEP, in the absence of other HIV prevention methods, is important for maintaining a seronegative HIV status.^9^, ^10^ However, measuring and understanding adherence to PrEP is complex as it relies on the simultaneous understanding of both PrEP use and sexual activity.^11^

Adherence to a pharmaceutical regimen refers to ‘the process by which patients take their medication as prescribed’, and is comprised of treatment initiation (when the patient takes their first dose), implementation (the extent to which a patient's actual dosing corresponds to the prescribed dosing regimen) and persistence (the length of time between initiation and the last dose).^12^ The determinants of suboptimal adherence may differ across these three processes, and hence may be amenable to different forms of intervention. Furthermore, while evidence‐based interventions exist for optimizing ARV medication prescribed as treatment,^13^ these may not translate directly to settings where ARVs are prescribed as prophylaxis—particularly when ‘optimal’ adherence will depend on the extent to which an individual engages in risk behaviours and the PrEP regimen followed, which itself may vary over time.

The aim of this study, therefore, was to gain an in‐depth understanding of the experiences and contextual factors that act as barriers and facilitators for the initiation, implementation and persistence with PrEP among individuals accessing it through the NHS in Wales.

## 2 MATERIALS AND METHODS

### 2.1 Study design and theoretical framework

We conducted a qualitative semistructured interview study of men who have sex with men (MSM) PrEP users in Wales. An interpretivist theoretical perspective was adopted, with the aim of understanding the subjective experiences of individuals through inductive reasoning.

### 2.2 Participant selection

Participants were individuals receiving TDF‐FTC as HIV PrEP through the NHS in Wales (a comprehensive, publicly funded health service)^14^ and participating in an ecological momentary assessment (EMA) study investigating PrEP use and sexual behaviour over time.^15^ Participants were approached consecutively upon completion of the EMA study. Those approached were sent study information via e‐mail, with SMS text message reminders sent to those who did not respond within 2 weeks. As an acknowledgement for their time, participants were offered a £20 gift voucher (with participants aware of this at the point of study approach).

We aimed to include between 20 and 30 participants in total, with this sample size informed by the information power model and taking into consideration the relatively narrow aims of the research, the identification of well‐defined strata (i.e., those who continued taking PrEP and those who discontinued), a theoretically informed topic guide (see below) and the strong emphasis placed on building trust and rapport with participants.^16^

### 2.3 Setting and data collection

All participants took part in semistructured interviews using the online video platform Zoom®. Participants were supplied with an individual meeting ID and password (available to only the researcher and participant), gave informed consent before the interview was conducted and consent was audio‐recorded. Consent procedures for the first four interviews were double‐checked by F. W. Interviews were conducted on a one‐to‐one basis, with the aim for them to last 30–60 min. The ABC taxonomy for describing and defining adherence to medications^12^ and components of the theory of planned behaviour^17^ were used to inform the topic guide. Questions were also asked covering the relationship between PrEP use and sexual behaviour, in addition to levels of support around PrEP use, and the perceived impact that PrEP has had on the lives of interviewees. The topic guide was reviewed and developed collaboratively amongst the research team and also with a stakeholder group. Field notes were taken during and after the interviews. Field notes taken during the interviews were primarily used as prompts to probe responses given by participants. See the Supporting Information Material for the topic guide.

Interviews were audio‐recorded and data were transcribed verbatim by a professional transcription service.

### 2.4 Data analysis

All transcripts were checked against the recording for accuracy by DG and anonymized. We conducted reflexive thematic analysis, outlined by Braun and Clarke, to analyse our interview data.^18^, ^19^ Following familiarization with the data, codes were developed by inspecting transcripts line by line, with an initial coding framework developed by D. G. Double coding was supported by coauthors F. W. and A. W. for the first four interviews to agree on the initial coding framework, accounting for alternative perspectives and subsequently by F. W. for a further three interviews to assess coding consistency. The initial coding framework was refined in response to input from F. W. and A. W., and a revised framework was shared amongst the research team and stakeholder group for further input. Themes were developed using the ‘One Sheet of Paper’ or ‘OSOP’ technique^20^ and were reviewed, refined and subsequently named. Direct participant quotes are presented with a Participant Identification number (PID) and these may include language that some readers may find triggering or offensive.

The analysis was supported by the qualitative data management software NVivo version 12.^21^

### 2.5 Research team and reflexivity

Interviews were conducted by the lead author D. G. D. G. is a postdoctoral research fellow and the chief investigator of the study. He has undertaken training in conducting and analysing qualitative interviews. D. G. is a 34‐year‐old White heterosexual cis‐gender male, with no lived experience of taking PrEP. D. G. was involved in the recruitment or follow‐up of all participants enrolled in the wider cohort.^15^

While similarly aged as the majority of interviewees, there is a risk that the differing sexual orientation of the interviewer and interviewees, in addition to the interviewer never haven taken PrEP, may have resulted in lower‐quality interview data through a lack of insight and shared experience. We attempted to minimize this through a team‐based approach to data analysis that allowed a wider range of perspectives to influence both the topic guide and analysis. Furthermore, by conducting follow‐ups with interviewees during their participation in the larger EMA study, the interviewer was able to gain trust and build rapport with interviewees before the interviews took place.

## 3 RESULTS

### 3.1 Participants

Thirty‐eight individuals were approached to take part in an interview. No response was received from 13, three declined participation and one who agreed did not turn up to the interview. In total, 21 participants were interviewed between 13 May 2020 and 6 November 2020. Interviews lasted 25–63 min (median duration 39 min). Participants were all cis‐gender males who exclusively had sex with other men. The majority were White British, the median age was 34 years (IQR: 27–43 years) and all except one adopted a daily PrEP regimen (with one participant taking event‐based PrEP). Table 1 highlights that the participants interviewed were broadly representative of those included in the larger cohort study, with a slight underrepresentation of those in full‐time employment at the time they entered the cohort study. Five interviewees had discontinued PrEP during the course of the cohort study.

**Table 1:** Characteristics of the interviewed participants (at the point of recruitment into the cohort)

	Interviewed [*N* = 21]		Approached and not interviewed [*N* = 17]		Not approached [*N* = 22]		Overall [*N* = 60]	
Variable	*n*	%	*n*	%	*n*	%	*n*	%
Sex								
Male	21	100.0	17	100.0	22	100.0	60	100.0
Gender								
Cis‐gender	21	100.0	17	100.0	22	100.0	60	100.0
Ethnicity								
White British	20	95.2	14	82.4	19	86.4	53	88.3
White European	1	4.8	1	5.9	2	9.1	4	6.7
White	0	0.0	1	5.9	0	0.0	1	1.7
African	0	0.0	1	5.9	0	0.0	1	1.7
White and Black African	0	0.0	0	0.0	1	4.5	1	1.7
Employment status								
Full‐time employed	11	52.4	16	94.1	15	68.2	42	70.0
Part‐time employed	3	14.3	0	0.0	3	13.6	6	10.0
Casual hours	4	19.0	1	5.9	1	4.5	6	10.0
Retired	2	9.5	0	0.0	2	9.1	4	6.7
Full‐time education	1	4.8	0	0.0	0	0.0	1	1.7
Not working	0	0.0	0	0.0	1	4.5	1	1.7
Education level								
Educated to degree level or equivalent	11	52.4	10	58.8	8	36.4	29	48.3
Educated to A‐levels or equivalent	7	33.3	6	35.3	5	22.7	18	30.0
Educated to general certificate of secondary education‐level (A^a^–C grades) or equivalent	3	14.3	1	5.9	9	40.9	13	21.7
PrEP status at recruitment into the cohort								
Starting PrEP for the first time (at recruitment)	4	19.0	5	29.4	2	9.1	11	18.3
Previously used PrEP	17	81.0	12	70.6	20	90.9	49	81.7
PrEP regimen								
Daily	20	95.2	16	94.1	21	95.5	57	95.0
Event‐based	1	4.8	1	5.9	1	4.5	3	5.0
Relationship status								
Single	17	81.0	12	70.6	17	77.3	46	76.7
In a relationship	3	14.3	5	29.4	4	18.2	12	20.0
Married	1	4.8	0	0.0	1	4.5	2	3.3
Sexual orientation								
Gay man	20	95.2	15	88.2	21	95.5	56	93.3
Bisexual	1	4.8	1	5.9	1	4.5	3	5.0
Pansexual	0	0.0	1	5.9	0	0.0	1	1.7
Sexual preference								
Has sex exclusively with men	21	100.0	17	100.0	21	95.5	59	98.3
Has sex with both men and women	0	0.0	0	0.0	1	4.5	1	1.7
Chronic health condition/s^a^								
At least one comorbid health condition	9	42.9	7	41.2	11	50.0	27	45.0
Asthma/respiratory condition^a^	3	14.3	3	17.6	3	13.6	9	15.0
Mood disorder/mental health condition^a^	4	19.0	0	0.0	2	9.1	6	10.0
Digestive tract condition^a^	2	9.5	2	11.8	2	9.1	6	10.0
Other condition^a^	3	14.3	4	23.5	8	36.4	15	25.0
	**Median**	**IQR**	**Median**	**IQR**	**Median**	**IQR**	**Median**	**IQR**
Age of participant	34	27–43	35	28–43	37	31–51	36	28–46

Abbreviations: IQR, interquartile range; PrEP, pre‐exposure prophylaxis.

^a^ Participants may have more than one health condition.

**Figure 1:**
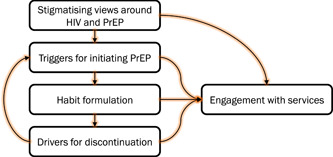
Thematic map underpinning key aspects of men who have sex with men PrEP users' experiences in Wales. PrEP, pre‐exposure prophylaxis

Figure 1 summarizes the themes developed as part of this study, and Table 2 illustrates a thematic matrix for the first theme.

**Table 2:** Example of thematic matrix underpinning qualitative analysis (Theme 1: Triggers for initiating PrEP)

Initial coding	Subtheme	Example of direct quote
HIV risk perceptions	A recognition that they were at risk of acquiring HIV was a key trigger for PrEP initiation. This was sometimes influenced by others or a standout incident (e.g., STI diagnosis or PEP use). It was also not necessarily the physical effects of having HIV, but the stigma issues and the impact that has on mental health.	PID 11: *Not for the, the illness itself, but the, just the way it affected his (ex‐partner who was living with HIV) mental health. Um, so I really wanted to be on PrEP because if I ever um, transmitted HIV, I would, I would fear, falling apart from stigma reasons*.
Partner's influence		
Advice from healthcare professionals		PID 31: *I'd just been to clinic… to get a check in relation to…some symptoms of STIs…, and as a result of a consultation with the consultant, she suggested that I took PrEP*.
Influence of friends and partners		PID 2: *It was a friend of mine, … they got up to various things and, and they, he, he showed concern for stuff that I was doing and the places I was going, so he, he suggested it*.
STI or worrying sexual encounter key trigger		PID 25: *Um I had a incident where I had to go on PEP, um and then after that then I thought it was more something I needed to get on as, as a preventative measure, so I didn't have to do it again, because it wasn't very pleasant*
Personal choice	A key factor underpinning the decision to start PrEP was the individual taking ownership of the responsibility for reducing his risk of acquiring HIV. By doing this, there was an acknowledgement that they were also protecting others.	PID 10: *In the end, it's your decision, to go on PrEP or not. You know, it's your decision to stay safe or not, you know. Err if your friends are gonna say* ‘*Oh no, don't worry, you shouldn't take it, you're gonna be fine*’*err this is not gonna be a support you know, it's not gonna be supporting, err I don't think err they shouldn't have an interest, I think the decision should be just yours*.
Protect self	Ownership of the responsibility for reducing their risk could also be framed as the individual exerting an element of control over the uncertain nature of HIV transmission.	PID 55: *It is something I probably would have considered if I was single as well, um, just being sexually active with more than one partner, it seems like the kind of risk that PrEP would mitigate a little*.
Protect others		PID 8: *to protect myself and in protecting myself, protecting others, you know, as well. Erm, so it was just to investigate and I thought, yeah, that's, you know, a reasonable step I can take*.
Access to PrEP	While some individuals had heard about PrEP being available for online purchase (or available elsewhere), a driver for individuals initiating PrEP was its availability through the NHS in Wales, which was generally viewed as a more trustworthy source of both medication and advice, in addition to being free of charge.	PID 7: *Having access to the prescription and, er, and regular treatment, and regular, because I've got friends in London who's got to pay for this and they, they haven't got the programme we've got in Wales… when I look at my friends now, they're taking it, stopping taking it, taking it, as and when they've got the money and stuff like that, so, it's affected then… For me, though personally it was a good thing ‘cause I had access to the medication, I could see that I was getting that protection that which I needed…*
Advice from the internet		
Trust in information about PrEP		

Abbreviations: NHS, National Health Service; PEP, postexposure prophylaxis; PID, Participant Identification number; PrEP, pre‐exposure prophylaxis; STI, sexually transmitted infection.

### 3.2 Triggers for initiating PrEP

The recognition and acceptance that they were at risk of acquiring HIV was a key trigger for individuals initiating PrEP. Furthermore, self‐recognition of risk was not always the starting point for individuals seeking out PrEP. Partners, friends and clinicians highlighting risk behaviours, particularly after standout incidents (e.g., an STI diagnosis, a postexposure prophylaxis [PEP] prescription), was a key feature highlighted during interviews.

I had an incident where I had to go on PEP [post‐exposure prophylaxis], and then after that I thought it was more something I needed to get on as, as a preventative measure, so I didn't have to do it again, because it wasn't very pleasant. (PID 25, 20–30 years, continued on PrEP)

The uncertainty surrounding the risk of HIV transmission during a sexual encounter and associated anxiety motivated some individuals to initiate PrEP to reduce their risk and thus exert some control. While this control and ownership largely centred on their own risks, this also extended more widely to sexual partners and more generally to everyone considered to be at risk.

It is something I probably would have considered if I was single as well. Just being sexually active with more than one partner, it seems like the kind of risk that PrEP would mitigate a little. (PID 55, 20–30 years, discontinued PrEP)

Moreover, concerns around acquiring HIV were not always about the physical effects of the disease, but also related to the concerns around the stigma associated with living with HIV and the impact that this may have on their mental health.

Not for the illness itself, but just the way it affected his [ex‐partner who was living with HIV] mental health. So I really wanted to be on PrEP because if I ever transmitted HIV, I would fear falling apart from stigma reasons. (PID 11, 31+ years, continued on PrEP)

While some individuals had previously purchased PrEP online and others had heard about the availability of PrEP through other means (e.g., clinical trials in England), the availability of PrEP through the NHS in Wales was viewed favourably due to its legitimacy, ease of access (it was available without a cap on numbers or without signing up to a clinical trial from the outset) and it being available free of charge.

Having access to the prescription and regular treatment, because I've got friends in London who's got to pay for this, and they haven't got the programme we've got in Wales… When I look at my friends now, they're taking it, stopping taking it, taking it, as and when they've got the money and stuff like that. So, it's affected them… For me though, personally it was a good thing ‘cause I had access to the medication, I could see that I was getting that protection that I needed… (PID 7, 31+ years, continued on PrEP)

### 3.3 Habitual behaviour for achieving high levels of implementation

The formulation of habits, or integration of PrEP use into existing habits, was perceived by participants to be a vital component of successful PrEP‐taking behaviour. This had been reinforced by clinic staff, who emphasized the importance of taking PrEP every day around the same time.

The preference to take PrEP daily instead of adopting an alternative regimen seemed motivated by a greater trust in the evidence around daily PrEP, as well as the ability to make PrEP an automatic action, separate from sex, and thus enabling greater spontaneity.

Do you know, I've never thought of event based dosing, because I think it [PrEP taking and sex] would be too much planning, too much preparation, when sometimes it [sex] can be quite spontaneous. For me, that kind of planning is probably a little bit too much, and I think I'd rather just take it every day. But I think again, I'd probably panic, thinking have I taken the right doses at the right times, have I done it enough days before, have I done it enough days after? Just it'd be a lot more to think about. (PID 15, 20–30 years, continued on PrEP)

Establishing a routine from scratch involved a process of trial and error. Participants reported exploring different methods for remembering to take PrEP regularly, with clear demonstration of self‐regulatory processes when methods were unsuccessful. Preprogrammed alarms were highlighted as a support tool participants used to assist their memory, with these particularly helpful at times where other routines were disrupted.

The integration of PrEP into an existing routine was viewed as the simplest way to maintain regular PrEP use, with this approach also requiring a process of trial and error. In those already taking a daily medicine or supplement (e.g., vitamins), PrEP was typically added as ‘just another tablet’ to this routine.

I tried, putting the bottle by my bed, so that I would take it first thing in the morning, and then I realised that I didn't take a drink to bed with me, so I had nothing to take it with in the morning… and then what I settled on then, when I finally got it sorted was um I'd have it on my living room table, where I have my breakfast and um I have a cup of tea with my breakfast, so the last bit of my tea would be to take my PrEP. Yeah it was just building some sort of way for me to incorporate it into stuff that I already do, it wasn't something additional that I had to do. (PID 25, 20–30 years, continued on PrEP)

Furthermore, disruption of a routine was cited as a key reason accounting for missed doses, with the consequences of missed doses considered within the context of the individual's recent sexual behaviour.

### 3.4 Short‐ and long‐term drivers for discontinuation

Participants described situations where they entered relationships that they considered to be long term and monogamous, general periods of reduced sexual activity and side‐effects outweighing benefits as key reasons supporting their decision to discontinue PrEP. There was a general weighing up of the risks and benefits of continuing to take PrEP. The risk of acquiring HIV through sexual contact, and hence the need for PrEP, was viewed as transient by some participants and this led to some temporary pauses in PrEP use while HIV risk was perceived to be low.

I was with a long term partner, and a few months in, I saw it as a long term relationship, it wasn't an open relationship, so I stopped taking the PrEP then. (PID 8, 20–30 years, discontinued PrEP)

For participants who had persisted with PrEP, situations in which they may consider discontinuing in the future aligned closely with the enacted reasons stated above. This may imply a general view of being a PrEP user as an impermanent state.

Probably one of the only things that would stop me would be if it started to affect me, my health basically. So if it was sort of having that negative affect rather than a positive effect, so if it did start to effect something like liver function or joints or something like that badly, then I'd probably say it's time to stop it. (PID 6, 31+ years, continued on PrEP)

However, some participants did not see there being a potential future whereby PrEP would be unnecessary for them.

I think if I ended up meeting somebody else … And, you know, sort of went into a relationship or anything I'd still stick with it anyway because it's not doing any sort of harm with me at the moment… If I completely stop meeting with people, stop any sort of intercourse at all then maybe but … no. (PID 4, 31+ years, continued on PrEP)

### 3.5 Engagement in services

Monitoring carried out at sexual health clinics, both in respect to the impact PrEP was having on their body as well as HIV testing, provided both initial and ongoing reassurance to participants that PrEP worked and was not causing them harm. Furthermore, the regular STI screening was generally viewed as a positive, as any infections could be detected and treated, thus minimizing any onwards transmission.

You're clean*, ‘cos you regularly get tested with a clinic. (PID 54, 20–30 years, continued on PrEP)

*Direct participant quote using language that some readers may find triggering or offensive.

By attending their sexual health clinic for a PrEP consultation, which included discussing their sexual activity since their previous visit with a consultant, collecting a PrEP prescription and undergoing screening tests, the perception of sexual health clinic visits moved from a less negative and reactive setting to a more positive and proactive setting.

I feel like when I'm taking PrEP it makes me feel like I'm doing something that is taking care of myself. I'm making like a concerted effort to actually put my health first. (PID 40, 20–30 years, discontinued PrEP)

## 4 DISCUSSION

In this study, we found that the initiation of PrEP was triggered following a recognition of HIV risk and an ownership of the responsibility for reducing risk. High‐quality implementation of PrEP was perceived to be facilitated by integrating PrEP within existing routines. Furthermore, PrEP was not typically viewed as a life‐long intervention. Indeed, hypothesized and enacted discontinuation was driven by either changes in sexual behaviour or side‐effects considered to outweigh the benefits of PrEP. Finally, PrEP altered the ways in which individuals engaged with sexual health services. Central to most themes was the role that stigma played in decision‐making—be it an underlying trigger for initiating PrEP, PrEP being seen as an opportunity to reduce discrimination against people living with HIV, or concerns around the perception of other PrEP users and individuals' own PrEP use.

This is the first study to qualitatively investigate PrEP‐taking experiences, fully incorporating established medication adherence taxonomy.^12^ Participants in this study were exclusively White MSM, the majority of whom were following a daily PrEP regimen, and while this is largely representative of PrEP users in Wales (91% of PrEP in Wales is prescribed daily),^22^ the experiences described in this paper may not reflect those of other key populations or of PrEP users adopting other regimens (e.g., event‐based dosing), particularly those focussing on habit formulation and integrating PrEP into an existing routine. Interviews were conducted remotely. This approach has been remarked upon as reducing geographical constraints with regard to data collection and reducing some barriers towards participation (e.g., commitments that may make travelling to a face‐to‐face interview challenging). However, it has also been suggested that remote online interviews may also exclude certain populations (e.g., those without access to digital technology) and limit the ability for the researcher to build trust and rapport with a participant.^23^

Themes incorporating initiating and discontinuing PrEP have been described in previous work exploring the barriers to PrEP use.^24^, ^25^, ^26^ While habit formulation is an often‐encouraged strategy to ensure high levels of medication adherence,^27^ the automatic action developed by integrating PrEP into existing routines could be thought of as individuals engaging in ‘System 1’ thinking, whereby conscious deliberate motivational processes do not feature in decision‐making.^28^ In other settings, MSM in a Netherlands‐based study indicated that the choice of daily over event‐based PrEP was similarly driven by a preference for unplanned sex.^29^ Moreover, the use of tools to assist with regular PrEP use was a strategy also highlighted by gay and bisexual men interviewed about PrEP use in Australia.^30^

Translating our themes into interventions to optimize adherence to PrEP, it is clear that each process of medication adherence may be amenable to different forms of intervention. Education, motivation and peer‐based interventions may enhance PrEP initiation, particularly if they increase awareness of the availability of PrEP, highlight HIV and other sexual health risks and address stigma concerns. On the latter point, it is apparent from both the experiences described and language used within interviews that HIV and PrEP‐associated stigma is pervasive in this population. Regular monitoring and feedback of PrEP use and sexual behaviour may facilitate habit formulation while normalizing self‐reflection on sexual risk behaviours and thus ongoing PrEP necessity. The most effective interventions would likely be multimodal, such as those trialled and shown to be most effective in improving adherence and health outcomes in individuals taking other preventative medication.^31^, ^32^

## 5 CONCLUSION

In conclusion, this study highlights key experiences regarding the initiation, implementation and persistence with PrEP, in addition to describing how PrEP may enhance sexual well‐being and promote more positive engagement with sexual health services. These findings may be useful for informing PrEP policy‐making, expansion of current programmes and need exploration in other key populations.

## CONFLICT OF INTERESTS

D. G. and K. H. report receiving funding from Health and Care Research Wales during the conduct of this study. K. H. also reports a leadership role for Cardiff University on the Fast Track Cardiff & Vale leadership group. This group is a local branch of the Fast Track Cities initiative aiming at eradicating HIV by 2030. R. M. reports funding from the National Institute for Health Research during the conduct of this study. The remaining authors declare that there are no conflict of interests.

## ETHICS STATEMENT

The study was reviewed and approved by the Wales Research Ethics Committee 3 (reference number: 19/WA/0175).

## AUTHOR CONTRIBUTIONS

David Gillespie led the design, collected data, conducted analysis and drafted the manuscript. Fiona Wood commented on early drafts of the manuscript. Fiona Wood and Adam Williams double‐coded a subset of transcripts and assisted in finalizing the initial coding framework. All authors reviewed the initial thematic matrices, comments on drafts of the manuscript and approved the final version for publication.

## Supporting information

Supporting information.

## Data Availability

Data (thematic coding matrices for all developed themes) are available on request from the authors. Due to privacy/ethical restrictions, other data (e.g., full transcripts) are not available.
