# Supplementary material for: Experiences of men who have sex with men when initiating, implementing and persisting with HIV pre‐exposure prophylaxis
Source: Health Expect. 2022 Apr 14;25(4):1332–41. doi: 10.1111/hex.13446 (PMC9327834; doi:10.1111/hex.13446)
Supplement: Supplementary file 1 — Supporting information. [file HEX-25--s001.docx]

**Experiences of men who have sex with men when initiating, implementing, and persisting with HIV pre-exposure prophylaxis: Supplementary material**

**Qualitative Interviews: Interview Schedule**

*[Look out of opportunities to prompt or probe around the behavioural topics covered in the quantitative study: stigma; risk perception; attitude; perceived norms; perceived behavioural control; intention; self-regulatory processes; action planning; anticipated regret]*

**Opening:**

My name is Dave and I am the Lead Investigator for the DO-PrEP project.

I would like to ask you some questions about your experiences taking PrEP.

I hope this information will help better understand how people take PrEP and whether any additional support for PrEP users is required.

The interview should take about 30 to 45 minutes.

Are you able to respond to some questions at this time?

**Before I start, please can you confirm the following:**

1. **That you have read the participant information sheet for this study (version 1.1 dated 14/06/2019), and have had the opportunity to ask any questions.**
2. **That you understand that participation in voluntary and that you are free to withdraw at any time, without giving reason.**
3. **That you will be asked questions about your experiences of using PrEP through the NHS in Wales.**
4. **That you agree that the interview can be audio recorded.**
5. **That you agree that anonymised quotations can be used in reports, presentations, and publications.**

**Please can you confirm that you give consent to take part in this study by stating your name and today’s date.**

**For my records, the participant identification number is X.**

**Body:**

*I’d like to start off by asking about how you got started on your PrEP journey*

***Topic 1:***

- PrEP story – How did you come to first start taking PrEP?
  - What encouraged you to start taking PrEP?
  - Were there any things that held you back from starting to take PrEP?

*Now I’d like to move on to ask about your experiences of using PrEP day to day*

***Topic 2:***

- PrEP use – Can you describe the course of PrEP you were prescribed – for example, how, when, and for how long you were advised to take it?
  - How difficult or easy has it been to follow your PrEP course/s?
  - What have been the main things that have made the prescription work well for you?
  - What have been the main things that have made the prescription not work well for you?
  - What are the main things that motivate you to continue with the prescription?
  - Can you describe to what extent PrEP has changed your sex life and in turn if and how your sex life influences how you take PrEP?
    - Can you describe whether and how PrEP influences your sex life?
  - Has there ever been a time where you have changed how you have taken your PrEP (from the way it was prescribed)? Can you describe what this change was and why you decided to make the change?

*Now I’d like to move on to ask about any experiences you have of stopping PrEP*

***Topic 3:***

- Stopping PrEP – Have you stopped taking PrEP for a week or more at any point during the study?

[If participant has stopped]:

- - How did this come to happen? Did you mean to do this or not?
  - What were the main reasons behind your decision?

[If participant hasn’t stopped]:

- - What are the main things that have motivated you to carry on?
  - What do you think would be the main reasons for you stopping in the future?

*Now I’d like to move on to ask about support you have (or would like to) receive for your PrEP use*

***Topic 4:***

- Support for taking PrEP
  - What kinds of support did you receive for starting PrEP?
  - What kinds of support have you received for taking your PrEP?
    - Personal support (e.g. friends, family, clinic staff, counsellors, others)
    - Non-personal support (reminders, etc.)
  - [If stopped]: What kinds of support did you receive when you decided to stop PrEP?
  - For all of the above – what kinds of support would you *like* to receive?

*I’d like to finish by asking about the impact that PrEP has had on your life*

***Topic 5:***

- Important outcomes – in what ways do you think taking PrEP has changed your life?
  - Good ways
  - Bad ways

*It has been a really interesting finding out about your PrEP experiences. Let me briefly summarise the information I have recorded during the interview…*

**Closing:**

I appreciate the time you’ve taken to give this interview. Is there anything else you think would be helpful for me to know about so I can use this to inform any additional support needs for people taking PrEP?

Thanks – I should have all of the information I need. Would it be alright to call you if I have any more questions?
